# High-Performance Hydrogen Gas Sensor Based on Pd-Doped MoS_2_/Si Heterojunction

**DOI:** 10.3390/s25154753

**Published:** 2025-08-01

**Authors:** Enyu Ma, Zihao Xu, Ankai Sun, Shuo Yang, Jianyu Jiang

**Affiliations:** School of Materials Science and Engineering, China University of Petroleum, Qingdao 266580, China; 2114010225@s.upc.edu.cn (Z.X.); s23140030@s.upc.edu.cn (A.S.); 2114010924@s.upc.edu.cn (S.Y.); z23140083@s.upc.edu.cn (J.J.)

**Keywords:** Pd-doped MoS_2_, sputtering, nanoparticles, heterojunction, gas sensor

## Abstract

High-performance hydrogen gas sensors have gained considerable interest for their crucial function in reducing H_2_ explosion risk. Although MoS_2_ has good potential for chemical sensing, its application in hydrogen detection at room temperature is limited by slow response and incomplete recovery. In this work, Pd-doped MoS_2_ thin films are deposited on a Si substrate, forming Pd-doped MoS_2_/Si heterojunctions via magnetron co-sputtering. The incorporation of Pd nanoparticles significantly enhances the catalytic activity for hydrogen adsorption and facilitates more efficient electron transfer. Owing to its distinct structural characteristics and sharp interface properties, the fabricated Pd-doped MoS_2_/Si heterojunction device exhibits excellent H_2_ sensing performance under room temperature conditions. The gas sensor device achieves an impressive sensing response of ~6.4 × 10^3^% under 10,000 ppm H_2_ concentration, representing a 110% improvement compared to pristine MoS_2_. Furthermore, the fabricated heterojunction device demonstrates rapid response and recovery times (24.6/12.2 s), excellent repeatability, strong humidity resistance, and a ppb-level detection limit. These results demonstrate the promising application prospects of Pd-doped MoS_2_/Si heterojunctions in the development of advanced gas sensing devices.

## 1. Introduction

Hydrogen gas (H_2_) holds significant potential in renewable energy transitions because it is clean, eco-friendly, renewable, and abundant. It has the capacity to improve air quality, mitigate global warming, and serve as a sustainable substitute for fossil fuels in both industry and transportation [1,2,3]. However, due to its high flammability in air within a concentration range of 4% to 75.6% [4], the real-time and precise detection of H_2_ levels is essential for practical applications. In the past few decades, numerous resistive hydrogen sensors based on metal oxide semiconductor materials have been studied, including In_2_O_3_, Pd/V_2_O_5,_ and SnO_2_ nanofibers [5,6,7]. However, these oxides usually require high operating temperatures above 300 °C. This not only results in higher power consumption, but also raises safety concerns due to hydrogen becoming highly flammable at elevated temperatures [8,9,10,11,12,13]. To address this challenge, it is crucial to develop H_2_ sensors capable of detecting trace amounts of hydrogen with rapid response at room temperature.

Two-dimensional (2D) materials, such as graphene, phosphorene, and transition metal dichalcogenides (TMDs), have garnered considerable attention as promising candidates for next-generation electronic devices, thanks to their exceptional electrical and mechanical characteristics and their large specific surface areas [14,15,16,17]. Among TMDs, MoS_2_ is particularly notable for its layered structure, where strong covalent bonds connect atoms within individual layers, while adjacent layers are held together by comparatively weak van der Waals forces. This structural arrangement increases the specific surface area, facilitating better contact with gas molecules. Furthermore, MoS_2_ exhibits an adjustable band gap between 1.2 and 1.9 eV, which can be tuned by varying the thickness of its layers [18]. Various studies have explored the use of few-layered MoS_2_ for gas sensing, demonstrating its capability to detect NH_3_, NO, H_2_O, and many other chemical vapors [19,20,21,22,23,24]. However, its basal planes are electrochemically inert and poorly conductive, which limits its ability to detect nonpolar gases like H_2_ [25]. Fortunately, structural imperfections—such as defects, vacancies, and dangling bonds—especially at edge sites or introduced during fabrication, can serve as active centers for gas adsorption and charge transfer [26].

To overcome the limitations of inert basal planes, recent research has explored the integration of noble metal catalysts to enhance surface reactivity and gas-sensing performance [27,28,29]. Among them, Pd stands out as an efficient catalyst for H_2_ detection. For example, Jaiswal et al. fabricated Pd-decorated MoS_2_ hybrid films, achieving enhanced hydrogen response due to nanoscale structural modulation [28]. Similarly, Baek et al. deposited Pd onto MoS_2_ films prepared by solution methods. The Pd catalyst facilitated H_2_ dissociation and spillover onto the sensing layer, resulting in superior sensing behavior [29]. Despite these promising results, achieving uniform distribution and controlled loading of noble metals in MoS_2_-based sensors remains a challenge. Effective H_2_ dissociation relies heavily on optimizing both the surface coverage and Pd content. To address this, magnetron co-sputtering has been adopted, enabling precise control of Pd nanoparticles doping while ensuring high coverage, uniformity, and crystallinity of the MoS_2_ films [30,31]. Due to interfacial effects, the heterojunction formed by sputtering MoS_2_ onto a silicon substrate is expected to enhance its gas sensing performance further. Wu et al. reported the construction of MoS_2_/Si nanowire array heterojunctions that achieved ultrahigh sensitivity for NO detection at room temperature, attributed to charge trapping and interfacial modulation effects [32]. Moreover, MoS_2_/Si heterojunctions were used as hydrogen sensors, but exhibited extremely long response times of approximately 443.5 s, which severely limits their practical application [33]. This highlights the need for optimizing the interfacial structure to improve performance. However, to the best of our knowledge, there are currently no reports on hydrogen sensors based on Pd-doped MoS_2_/Si heterojunctions. This indicates a research gap in simultaneously utilizing Pd nanoparticle doping and heterostructure engineering under room temperature conditions.

The aim of this study is to develop a high-performance hydrogen gas sensor operating at room temperature, based on Pd-doped MoS_2_/Si heterojunctions prepared via magnetron co-sputtering. By precisely controlling both the doping of Pd nanoparticles and the formation of the heterojunction interface, the sensor can synergistically improve hydrogen adsorption, electron transfer, and dynamic sensing behavior. The fabricated Pd-doped MoS_2_/Si heterojunction exhibited an excellent H_2_ sensing response (6.4 × 10^3^%), faster response/recovery times (24.6/12.2 s), outstanding repeatability, strong resistance to humidity, and a ppb-level detection limit. This study delivers a promising strategy to the design and optimization of high-response hydrogen sensors, offering strong potential for next-generation gas sensor applications.

## 2. Materials and Methods

n-type silicon wafers with a (100) crystallographic orientation and a thickness of approximately 0.5 mm were employed. These single-crystal substrates, purchased from Suzhou Yancai Weina Technology Co., Ltd. (Nanfeng, China), had a resistivity of 1 × 10^5^ Ω·cm and a purity of 99.99%. The wafer, sliced into pieces measuring 10.0 mm × 10.0 mm, underwent sequential ultrasonic cleaning using ethanol, acetone, and deionized water. Pd-doped MoS_2_ thin films were grown on a Si substrate using the DC magnetron co-sputtering technique. MoS_2_ targets (purity 99.99%) were obtained from Zhongnuo Xincai Technology Co., Ltd. (Beijing, China), and Pd sources (purity 99.99%) were supplied by Beijing Global JinXin International Co., Ltd. (Beijing, China). During the co-sputtering process, high-purity Ar was employed as the sputtering gas under a chamber pressure of 1 Pa. The sputtering powers for Pd and MoS_2_ targets were set to 1.0 W and 10.0 W, respectively, and the deposition was carried out at a substrate temperature of 400 °C. Finally, Pd was deposited onto the doped MoS_2_ layer as the top electrode by magnetron sputtering using Ar gas at a pressure of 1 Pa and a sputtering power of 10 W, while indium (In) was manually applied to the rear side of the Si substrate using a soldering iron as the bottom electrode. Electrical measurements were performed by connecting the electrodes using 0.1 mm diameter copper wires.

**Figure 1:**
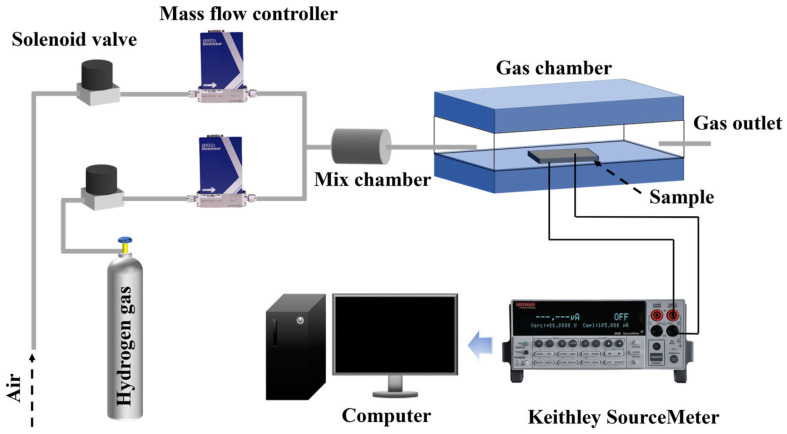
Schematic diagram of the gas flow system used for sensor device testing.

The film’s surface was analyzed using a scanning electron microscope (TESCAN MIRA LMS, Brno, Czech Republic). The crystalline structure of the thin films was examined using Bruker D8 X-ray diffraction (XRD). Raman spectroscopy (HORIBA, HR800, Kyoto, Japan), employing a 532 nm excitation wavelength, was utilized to investigate the Pd-doped MoS_2_ thin films. X-ray photoemission spectroscopy (Thermo K-Alpha spectrometer) was performed to determine the chemical composition. The optical bandgap was assessed with a Hitachi UH4150 spectrophotometer. Figure 1 illustrates the setup employed to investigate the gas sensing characteristics of the Pd-doped MoS_2_/Si heterojunction. The sensor device was positioned inside a sealed chamber, integrated with a gas flow system that included gas cylinders and mass flow controllers for gas sensing experiments. These mass flow controllers, connected to the chamber’s upper ports, precisely regulated the gas flow rates, enabling the creation of H_2_ at varying concentrations. The I–V characteristics of the sensor, under different gas conditions, were recorded using a Keithley 2400 digital source meter.

## 3. Results

### 3.1. Thin Film’s Characterization

**Figure 2:**
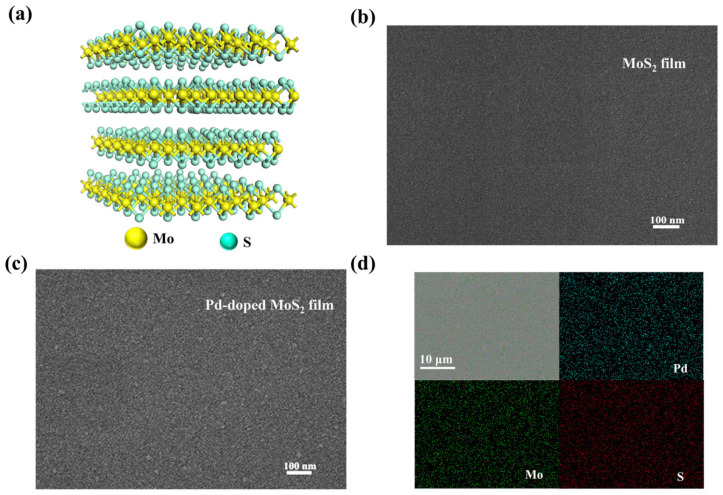
(**a**) Crystal structure of layered MoS_2_; (**b**) SEM surface images of MoS_2_ film; and (**c**) PD-doped MoS_2_ film. (**d**) Top-view SEM morphology and corresponding elemental distribution from EDS mapping of the Pd-doped MoS_2_ layers.

As shown in Figure 2a, bulk MoS_2_ possesses a layered crystalline architecture. This distinctive arrangement, coupled with the lack of dangling bonds between neighboring layers, enables the production of ultrathin MoS_2_ sheets characterized by smooth surfaces and large specific surface areas. To further investigate the crystal structure of the Pd-doped MoS_2_ thin film, XRD analysis was performed (Figure S1, Supplementary Materials). The Pd-doped MoS_2_ film exhibits distinct diffraction peaks at 14.4° and 51.3°, which correspond to the (003) and (018) crystal planes, respectively (PDF No. 74-0932), indicating good crystallinity and successful formation of the layered MoS_2_ structure. In addition, no new diffraction peaks were observed after Pd doping, suggesting that the doping concentration is relatively low and does not lead to the formation of any detectable secondary phases. Figure 2b,c show the SEM images of MoS_2_ films before and after Pd doping. Figure 2b displays the surface Structure of the pristine MoS_2_ film, revealing a smooth texture with no obvious grain boundaries. This observation suggests that the MoS_2_ thin film is uniformly deposited across the substrate. After Pd doping (Figure 2c), the surface becomes noticeably rougher, and clear grain boundaries appear. The increased surface roughness facilitates the introduction of more structural defects, thereby generating additional active sites for reaction with H_2_ molecules. Pd nanoparticles mainly occupy and interact with defect sites introduced into the MoS_2_ basal planes during sputtering, rather than acting on ideal, defect-free basal surfaces [34]. The microstructure and chemical composition of the Pd-doped MoS_2_ thin films were investigated using energy-dispersive spectroscopy (EDS). Figure 2d shows the elemental distribution map obtained from the film’s planar view. The Pd element is very uniformly distributed within the thin film, indicating that Pd nanoparticles have been successfully and homogeneously doped into the MoS_2_ thin film. Figure S2 (Supplementary Materials) presents the cross-sectional SEM image of the Pd-doped MoS_2_ film deposited on a Si substrate with a sputtering time of 1200 s. The layered structure of MoS_2_ is not clearly visible in the image. Instead, the film exhibits a distinctly crumpled morphology, which is markedly different from the typical flat MoS_2_ nanosheets and is likely attributed to the incorporation of Pd nanoparticles. The extended sputtering time was chosen to achieve better imaging contrast in SEM, and the measured film thickness is approximately 330.84 nm. However, the Pd-doped MoS_2_ film used in the actual gas sensing tests in this study was fabricated with a sputtering time of only 10 s. Considering a deposition rate of approximately 0.28 nm/s, and based on previous studies reporting each MoS_2_ layer to be about 0.7 nm thick [35], the film used in the tests is estimated to consist of about four layers, with a total thickness of approximately 2.8 nm.

**Figure 3:**
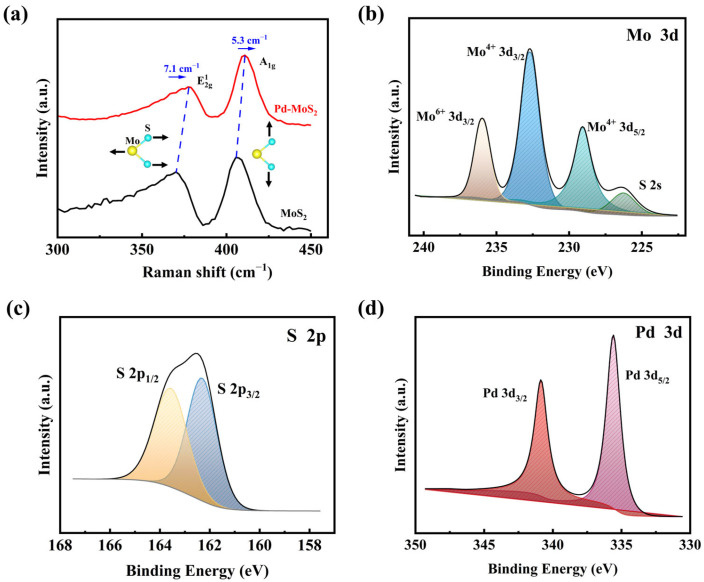
(**a**) Raman spectra of the prepared Pd-doped MoS_2_ and pristine MoS_2_ nanosheets. The XPS characterization of the Pd-MoS_2_ thin film: High-resolution XPS spectra of (**b**) Mo 3d, (**c**) S 2p, and (**d**) Pd 3d.

Figure 3a presents the Raman spectra for both MoS_2_ and Pd-doped MoS_2_ films, revealing two characteristic peaks that correspond to the $E_{2g}^{1}$ and A_1g_ vibrational modes of MoS_2_ [36,37]. $E_{2g}^{1}$ represents the in-plane vibrational mode parallel to the substrate, while A_1g_ corresponds to the out-of-plane mode. In Pd-doped MoS_2_, both peaks exhibit a blueshift, with $E_{2g}^{1}$ and A_1g_ shifting by 7.1 cm^−1^ and 5.3 cm^−1^, respectively, compared to pristine MoS_2_. This blueshift indicates that Pd doping introduces p-type characteristics. The elemental composition of Pd-doped MoS_2_ thin films was further characterized using XPS. The Mo 3d XPS spectrum is depicted in Figure 3b. The double peaks observed at 232.88 eV (Mo 3d_3/2_) and 229.08 eV (Mo 3d_5/2_) are indicative of the Mo^4+^ state, respectively. The additional peaks identified at 235.74 eV and 226.58 eV correspond to Mo^6+^ states (due to the oxidation of surface-adsorbed oxygen) and the S 2s. Figure 3c displays peaks located at 162.31 eV and 163.56 eV corresponding to the S 2p_3/2_ and S 2p_1/2_ orbitals. This result is consistent with the reported MoS_2_ crystal data [38]. Pd 3d XPS spectra (Figure 3d) show characteristic double peaks at 335.48 eV and 340.78 eV, corresponding to Pd 3d_5/2_ and Pd 3d_3/2_ states of metallic Pd nanoparticles [39]. The binding energies are consistent with metallic Pd, indicating that Pd is present in the form of nanoparticles. This confirms the successful doping of Pd nanoparticles into the MoS_2_ matrix, with a Pd concentration of 11.6 at% in the doped sample.

### 3.2. Gas Sensing Analysis

**Figure 4:**
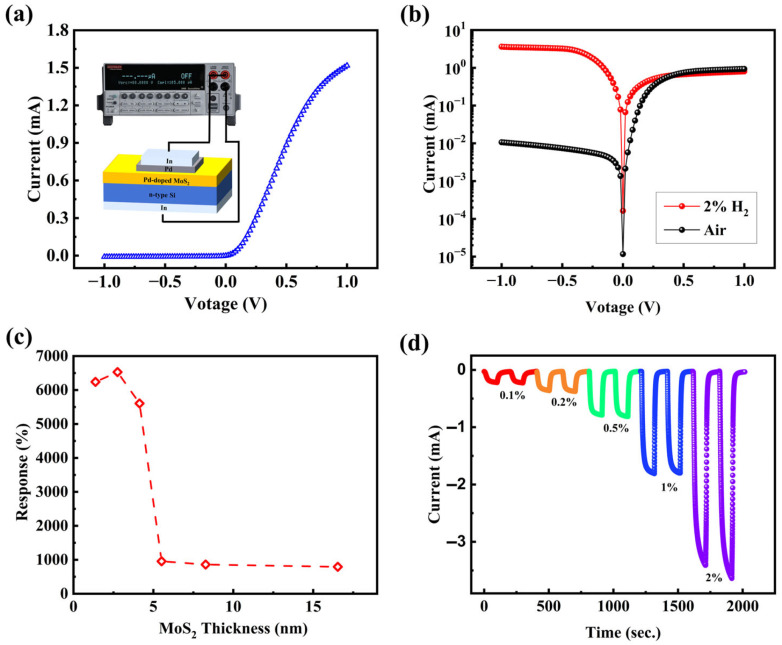
(**a**) I–V curve of the Pd-doped MoS_2_/Si heterojunction, with the inset providing a schematic diagram of the measurement setup. (**b**) I–V semilogarithmic plot of Pd-doped MoS_2_/Si heterojunction sensor exposed in air and 20,000 ppm H_2_, respectively. (**c**) Response of the MoS_2_ layers with different thicknesses in the MoS_2_/Si sensor. (**d**) Dynamic consecutive responses of the Pd-doped MoS_2_/Si transducer under various H_2_ concentrations at a bias voltage of −1.0 V and room temperature.

Figure 4a displays the characteristic I–V curve for the heterojunction, with an inset illustrating the measurement setup schematic. The curve exhibits pronounced rectifying behavior, achieving a rectification ratio of approximately 10^2^ at ±1.0 V, suggesting a junction forms at the Pd-doped MoS_2_/Si interface. Figure 4b shows semi-logarithmic I–V plots for Pd-doped MoS_2_/Si heterostructure devices, measured in air and H_2_ environments at room temperature. As illustrated in Figure 4b, a noticeable rise in the I–V curve in the negative voltage range occurs when the sensor device is exposed to H_2_ instead of air, indicating that the heterojunction exhibits a distinct response to H_2_ exposure. The sensor response was determined according to the following equation:

(1) $$sensor\;response=\;\frac{I_{H_{2}}}{I_{Air}}\;\times 100\%.$$

where *I_H2_* and *I_Ai_*_r_ represent the currents measured in hydrogen and air environments, respectively. A series of MoS_2_ films with varying thicknesses was fabricated, and their response to 20,000 ppm hydrogen concentration was evaluated (Figure 4c). The highest sensor response was observed for a MoS_2_ layer thickness of approximately ~2.8 nm. Previous research has indicated that the response of MoS_2_ thin films decreases significantly with increasing thickness (2–16 nm) [40], which is primarily attributed to the reduced surface-to-volume ratio, ultimately leading to lower response. All subsequent experiments and discussions are based on a 2.8 nm thickness. Figure S3 (Supplementary Materials) displays the I–V characteristics of the Pd-doped MoS_2_/Si heterojunction exposed to 20,000 ppm H_2_ under varying humidity levels of 50%, 60%, 70%, and 80% RH. As the humidity rises from 50% to 80%, the current measured at 1.0 V shows a slight decline, reflecting a 51% change in response. This reduction may be attributed to the adsorption of H_2_O molecules on the heterojunction surface, which potentially hinders the effective interaction between hydrogen molecules and Pd nanoparticles by limiting their contact area. As shown in Figure 4d, the dynamic response of the Pd-doped MoS_2_/Si was measured at the hydrogen concentrations spanning from 1000 ppm to 20,000 ppm. The current response exhibits a progressive increase in amplitude as the hydrogen concentration rises. Notably, the sensor exhibits a significant response performance even at the low concentration of 1000 ppm. The limit of detection (*LOD*) is determined using the following equation [41]:

(2) $$LOD=\frac{3\sigma_{noise}}{s}$$

where $\sigma_{noise}$ is the root mean square of noise and *s* is the slope of the response versus concentration plot. The *LOD* of the sensor is 360 ppb, demonstrating its great potential for applications in hydrogen detection. The repeatability of the Pd-doped MoS_2_/Si sensor was investigated under 10,000 ppm H_2_ at room temperature. According to Figure S4 (Supplementary Materials), the sensor exhibited a consistent dynamic response over 14 consecutive cycles. When alternately exposed to air and hydrogen, the device demonstrated a clear and consistent switching behavior, characterized by elevated current levels in air and suppressed currents in hydrogen. No significant variation in response was observed, indicating excellent repeatability during the switching process.

**Figure 5:**
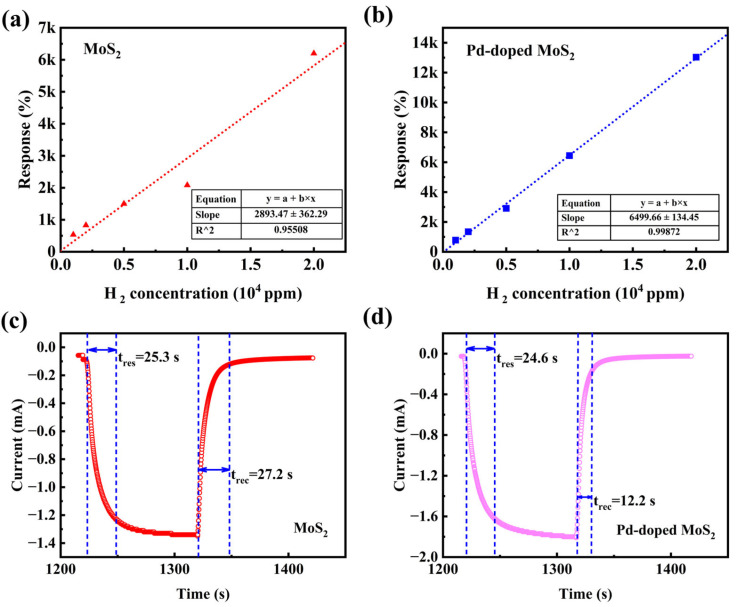
Relationship between responsiveness and hydrogen concentration of (**a**) MoS_2_ and (**b**) Pd-MoS_2_/Si devices. (**c**) Response curves of undoped MoS_2_ sensors under dynamic conditions toward 10,000 ppm H_2_ at RT. (**d**) Response curve of the Pd-doped MoS_2_/Si sensing devices at 10,000 ppm H_2_ under dynamic conditions at RT.

For comparison, we also evaluated the response of the undoped MoS_2_/Si sensor under different H_2_ concentrations. Figure 5a,b present the linear fitting curves of the response as a function of H_2_ concentration for both MoS_2_/Si and Pd-doped MoS_2_/Si devices. When the H_2_ concentration increases from 1000 ppm to 20,000 ppm, the response of the MoS_2_/Si device increases from 500% to 6200%, whereas the response of the Pd-MoS_2_/Si device increases from 800% to 13,000%. The sensor response exhibits a linear increase with increasing H_2_ concentration, and this linear fitting enables estimation of the sensor response at any H_2_ concentration within the range of 1000 ppm to 15,000 ppm. The calibration equations and corresponding correlation coefficients (R^2^) describing the relationship between H_2_ concentration and sensor response for both MoS_2_/Si and Pd-doped MoS_2_/Si devices are provided in Figure 5a,b. The linear fitting slope of the Pd-MoS_2_/Si device is significantly greater than that of the MoS_2_/Si device, indicating a notably enhanced response of the Pd-doped MoS_2_/Si sensor at each concentration level. Figure 5c shows the dynamic response and recovery behavior of the undoped MoS_2_/Si sensor toward 10,000 ppm H_2_ at room temperature, while Figure 5d presents the corresponding response curve of the Pd-doped MoS_2_/Si sensor under the same conditions. The response time and recovery time are defined as the specific intervals during which the sensor reaches 90% of its stable current level and returns to within 10% of its initial baseline value, respectively. The results at a hydrogen concentration of 10,000 ppm indicate that the Pd-doped MoS_2_/Si sensor exhibits significantly enhanced H_2_ sensing performance compared to the undoped MoS_2_ sensor. Specifically, the undoped MoS_2_ sensor shows a response of 2100% with a relatively slow response/recovery time of 25.3 s/27.2 s, whereas the Pd-doped MoS_2_/Si sensor achieves a much higher response of 6400% with faster response/recovery times of 24.6 s/12.2 s. This improvement is attributed to the higher density of active sites resulting from Pd doping, which promotes greater hydrogen adsorption, as well as the enhanced electrical conductivity, which facilitates more efficient electron transfer. As a result, both the response magnitude and response/recovery speed are considerably improved.

**Figure 6:**
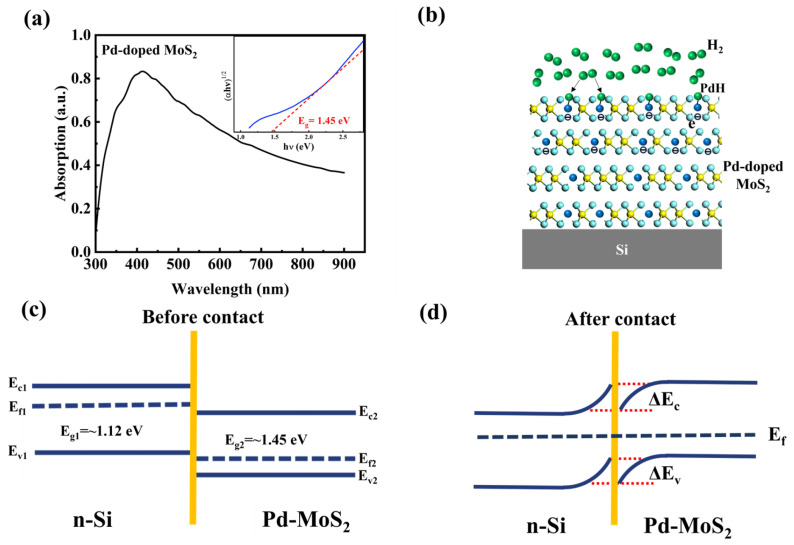
(**a**) The UV absorption spectrum of Pd-doped MoS_2_ films; (**b**) Schematic diagram of the electron generation process in the Pd-doped MoS_2_/Si device; (**c**,**d**) Band-energy diagram of the Pd-MoS_2_/Si heterojunction prior to and following contact, respectively.

Based on the ultraviolet-visible absorption spectrum (UV) shown in Figure 6a, the structure becomes denser after Pd doping, allowing for greater light absorption. As a result, its absorbance is significantly improved compared to that of pure MoS_2_ (Figure S5, Supplementary Materials). The bandgap of the Pd-doped MoS_2_ thin film is approximately 1.45 eV, which is narrower than that of pure MoS_2_. This narrower bandgap makes it easier for electrons to be excited to the conduction band, thereby enhancing the response, which is consistent with previous studies [42]. Figure 6b illustrates the schematic diagram of electron generation and transport in the Pd-doped MoS_2_/Si device. When the Pd-doped MoS_2_ thin film is exposed to a H_2_ environment, the Pd nanoparticles distributed on or near the surface of MoS_2_ act as active catalytic sites and react with hydrogen molecules to form PdH_x_ [43]. During this process, a large number of electrons are released from the PdH_x_ and injected into the MoS_2_ thin film, thereby compensating the originally existing hole carriers and leading to a reduction in hole concentration. As the hole concentration decreases, the Fermi level of the MoS_2_ thin film shifts upward, moving closer to the conduction band. Figure 6c illustrates the interfacial electronic band structure formed between MoS_2_ and Si. According to the literature [44], the band parameters of n-type Si are as follows: work function W = 5.0 eV, conduction band minimum E_C_ = 4.05 eV, valence band maximum E_V_ = 5.17 eV, and bandgap E_g_ = 1.12 eV. Owing to the relatively elevated Fermi level of the n-type Si substrate at the interface, electrons migrate from the Si to the Pd-doped MoS_2_ layer. This migration persists until equilibrium is established between the Fermi levels of both materials, ultimately forming the Pd-MoS_2_/Si heterojunction in Figure 6d. Electrons released by the H_2_ reaction are injected into the Pd-MoS_2_ film under reverse bias and further transferred to the Pd-doped MoS_2_/Si interface. The accumulation of electrons at the interface causes an upward shift in the E_F_ of Pd-MoS_2_, bringing it closer to E_C_. The reduction in interfacial energy barrier height significantly enhances the reverse current of the heterojunction [45]. Consequently, the Pd-doped MoS_2_/Si heterojunction exhibits unique advantages in the development of high-performance H_2_ sensors with superior gas response.

Table 1 compares the performance of the H_2_ sensors fabricated in this work with other related devices. The novelty of this work lies in the successful uniform doping of Pd nanoparticles into the MoS_2_ thin film using magnetron co-sputtering technology. In comparison, Pd-doped MoS_2_/Si heterojunctions exhibit excellent H_2_ sensing performance at room temperature, particularly demonstrating a higher H_2_ response along with significantly shorter response and recovery times. The enhanced sensing performance of the Pd-doped MoS_2_/Si heterojunction mainly stems from several key contributing factors: (i) The layered structure of MoS_2_ possesses a large specific surface area, which not only increases the contact area with gas molecules but also facilitates the regulation of structural defects and surface active sites, thereby enhancing gas adsorption and reaction performance. (ii) Appropriately doped Pd nanoparticles expose more surface active sites for hydrogen molecule adsorption and provide additional electron transport pathways, significantly improving the adsorption rate of hydrogen and the efficiency of electron transfer. (iii) Due to the sharp interface formed between Pd-doped MoS_2_ and Si, a significant energy barrier is established at the heterojunction, which effectively suppresses the background current under equilibrium conditions. When electrons are injected into the Pd-MoS_2_ layer during H_2_ exposure, the accumulation of charge leads to a reduction in the interfacial barrier height, thereby significantly increasing the reverse current. This dynamic modulation of the barrier enhances the device’s sensitivity to hydrogen, resulting in a more pronounced relative change in electrical response. However, to meet the demands of practical applications for faster response and shorter recovery times, there remains room for improvement in the dynamic performance of the sensor. Based on this, two effective optimization strategies are proposed in this work: (1) Structuring MoS_2_ into vertically aligned or edge-enriched nanoflake morphologies can substantially increase the number of exposed active edge sites and reduce gas diffusion pathways, thereby accelerating the adsorption and desorption kinetics of hydrogen molecules. Vertically aligned MoS_2_ films have been reported to exhibit significantly enhanced sensitivity and faster switching behavior compared to their basal-plane counterparts in gas sensing applications [46]. (2) Applying mild O_2_ plasma treatment can effectively passivate deep-level trap states by filling sulfur vacancies with oxygen-containing species, which leads to improved carrier mobility and a substantial reduction in response and recovery times. These strategies are expected to significantly enhance the sensing speed and real-time performance of the Pd-doped MoS_2_/Si device.

## 4. Conclusions

In summary, the Pd-doped MoS_2_/Si heterojunction was successfully developed, exhibiting excellent H_2_ detection performance. The deposited MoS_2_ films possess high crystallinity with uniformly distributed nanoparticles, while Pd doping exposes more active sites for H_2_ molecule adsorption and enhances the efficiency of electron transfer. As a result, the Pd-doped MoS_2_/Si heterojunction device achieves a high response of 6.4 × 10^3^%, fast response and recovery times of 24.6/12.2 s, excellent repeatability, strong humidity resistance, and a ppb-level detection limit at room temperature. These findings demonstrate the potential of Pd-doped MoS2/Si heterojunctions for high-performance H_2_ sensor applications.

## Figures and Tables

**Table 1:** Performance comparison in the present work with other H_2_ sensors.

Materials	Fabrication Method	H_2_ Concentration (ppm)	Response (%)	Response/Recovery Times (s)	Temperature	Ref.
MoS_2_ flake	CVD	10,000	1	14.3/137	RT	[47]
Pd–SnO_2_/MoS_2_	Hydrothermal method	5000	18	30/19	RT	[48]
Pt-WO_3_	PVD	5000	68	~	110 °C	[49]
Pt/Pd-ZnO	PLD	10,000	58	5/76	100 °C	[50]
MoS_2_/GaN	Sputtering	10,000	150	~	157 °C	[51]
Pt-MoS_2_	Hydrothermal method	2000	75	150/370	100 °C	[52]
Pd-doped MoS_2_/Si	Sputtering	10,000	6400	24.6/12.2	RT	This work

## Data Availability

The authors confirm that the data supporting the findings of this study are available within the article.

## Supplementary Materials

The following supporting information can be downloaded at: https://www.mdpi.com/article/10.3390/s25154753/s1. Figure S1: X-ray diffraction (XRD) θ–2θ pattern of the Pd-doped MoS_2_ films; Figure S2: Cross-sectional SEM micrographs of Pd-doped MoS_2_ layers grown on silicon wafers with a sputtering time of 1200 s; Figure S3: I–V characteristics of the Pd-doped MoS_2_/Si heterojunction exposed to 20,000 ppm H_2_ under varying humidity levels of 50%, 60%, 70%, and 80% RH; Figure S4: Repeatability of the fabricated Pd-doped MoS_2_/Si sensor at room temperature when exposed to 10,000 ppm H_2_; Figure S5: The UV absorption spectrum of MoS_2_ films.
