# Supplementary material for: High-Performance Hydrogen Gas Sensor Based on Pd-Doped MoS2/Si Heterojunction
Source: Sensors (Basel). 2025 Aug 1;25(15):4753. doi: 10.3390/s25154753 (PMC12349308; doi:10.3390/s25154753)
Supplement: Supplementary file 1 [file sensors-25-04753-s001.zip › sensors-3773285-supplementary.pdf]

Supplementary Materials

High-Performance Hydrogen Gas Sensor Based on Pd-Doped MoS_2/Si Heterojunction

Enyu Ma *, Zihao Xu, Ankai Sun, Shuo Yang and Jianyu Jiang

School of Materials Science and Engineering, China University of Petroleum, Qingdao 266580, China.

* Correspondence: 2214010105@s.upc.edu.cn

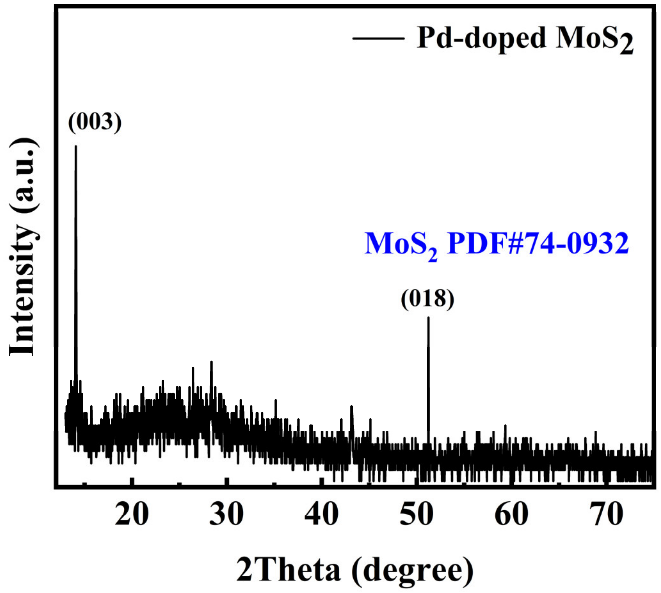

Figure S1. X-ray diffraction (XRD) θ–2θ pattern of the Pd-doped MoS_2 films.

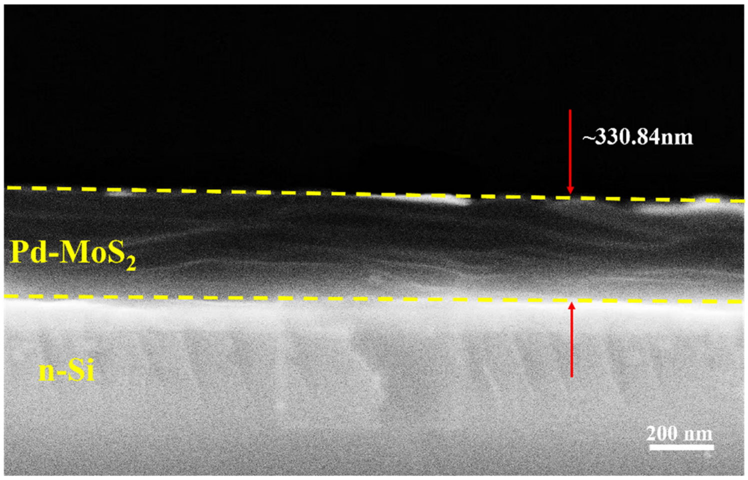

Figure S2. Cross-sectional SEM micrographs of Pd-doped MoS_2 layers grown on silicon wafers with a sputtering time of 1200 s.

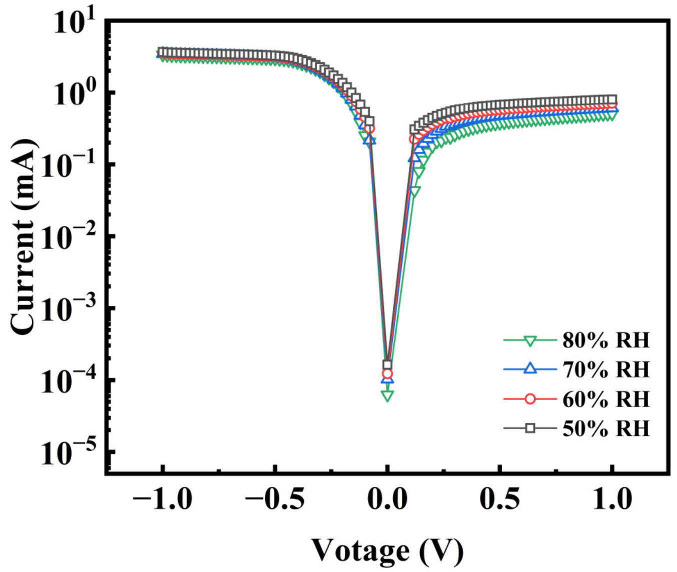

Figure S3. I–V characteristics of the Pd-doped MoS_2/Si heterojunction exposed to 20,000 ppm H_2 under varying humidity levels of 50%, 60%, 70%, and 80% RH.

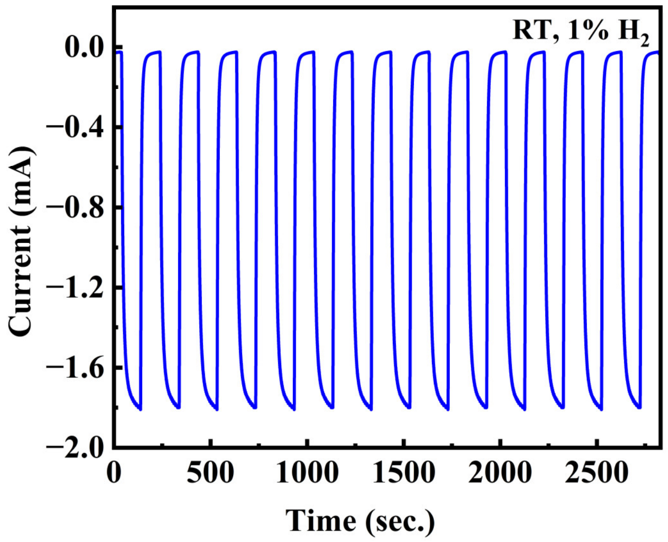

Figure S4. Repeatability of the fabricated Pd-doped MoS_2/Si sensor at room temperature when exposed to 10,000 ppm H_2.

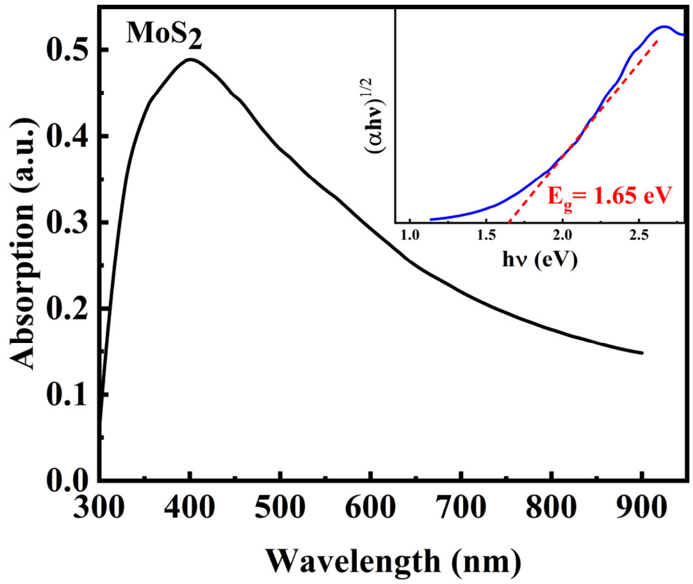

Figure S5. The UV absorption spectrum of MoS_2 films.
